# Progress in the Application of the Residual SYNTAX Score and Its Derived Scores

**DOI:** 10.31083/j.rcm2503080

**Published:** 2024-03-01

**Authors:** Xinjun Lin, Zhibin Mei, Wei Ji, Yaoguo Wang, Chaoxiang Xu

**Affiliations:** ^1^The Second Clinical College of Fujian Medical University, 362000 Quanzhou, Fujian, China; ^2^Department of Cardiology, the Second Affiliated Hospital of Fujian Medical University, 362000 Quanzhou, Fujian, China

**Keywords:** residual SYNTAX score, residual functional SYNTAX score, rational revascularization, coronary artery disease, prognosis

## Abstract

The residual SYNTAX score (rSS) is employed for the quantification of residual coronary lesions and to guide revascularization. rSS can be combined with other examinations to evaluate the severity of vascular disease and play an evaluative and guiding role in various scenarios. Furthermore, combining rSS with other indicators, benefits prognosis evaluation, and rSS-derived scores have been increasingly used in clinical practice. This article reviews the progress in the clinical application of rSS and its derived scores for complex coronary arteries and other aspects, based on relevant literature.

## 1. Introduction

Coronary artery disease (CAD) is a prevalent cardiovascular disorder associated with significant mortality rates [1], and complex coronary artery lesions are the focus of CAD treatment. With the popularization and improvement of percutaneous coronary intervention (PCI) technology and related device materials, PCI has increasingly become a frequent and important means of managing CAD. However, the occurrence of major adverse cardiovascular events (MACE) in certain patients undergoing PCI remains high [2]; therefore, choosing a reasonable treatment strategy for CAD is particularly important.

**Fig. 1.**
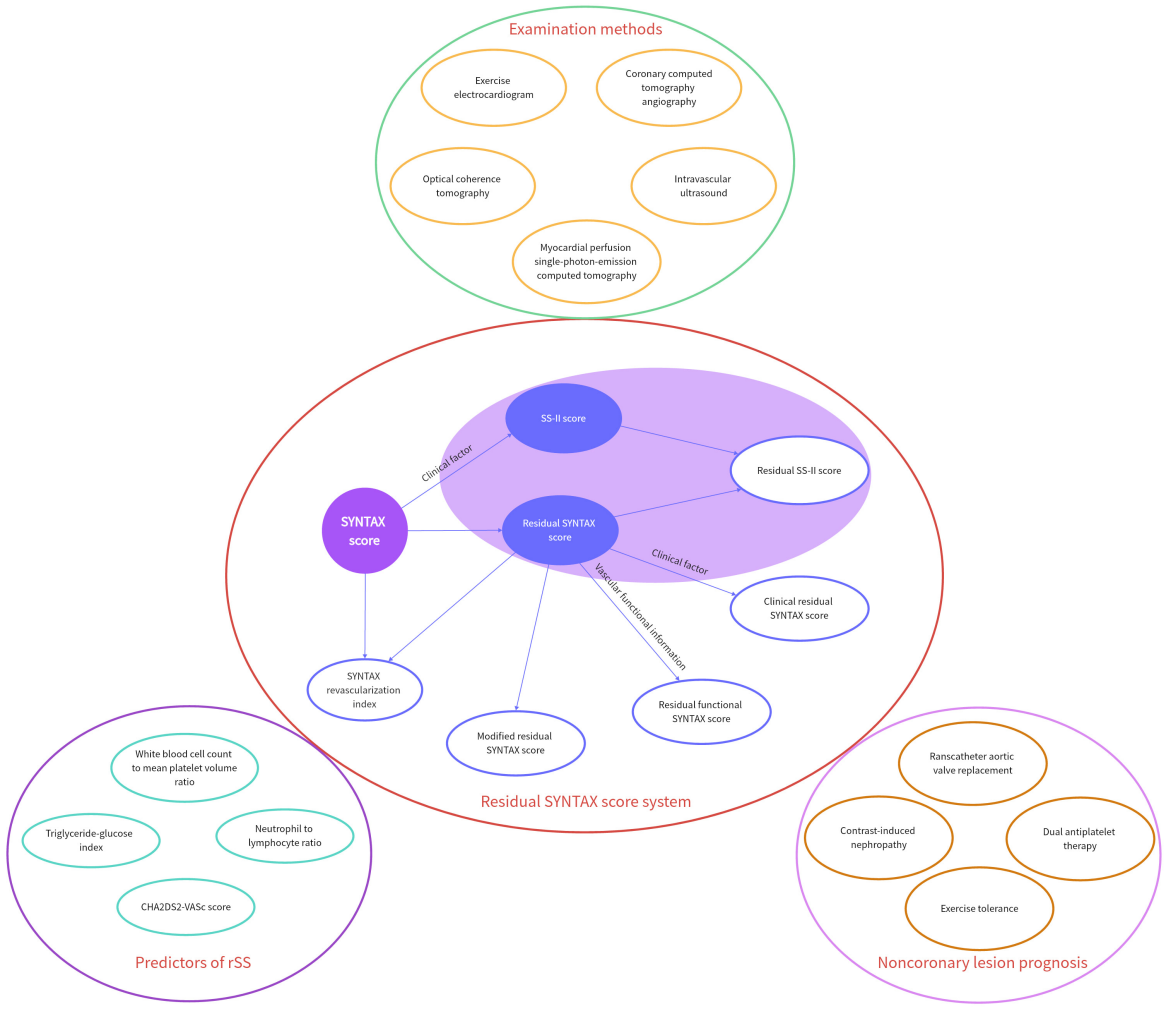
**Mind map of residual SYNTAX score (rSS) and its derived scores**.

Sianos *et al*. [3] proposed the SYNTAX score (SS) in 2005, based on the SYNTAX study. SS can predict patients’ prognoses after PCI or coronary artery bypass graft (CABG) and evaluates untreated coronary vessels. Vessels after interventional treatment and untreated vessels have significant anatomical differences; notably, the operator’s surgical experience, surgical strategy, and surgical environment contribute to the differences. SS cannot effectively evaluate this difference; therefore, it cannot fully reflect the improvement of coronary vessels. With the continuous improvement of the SS system, the residual SYNTAX score (rSS) has been proposed to quantitatively measure the extent of residual coronary artery stenosis after PCI. After continuous clinical exploration, the rSS system and its application range have expanded. In addition, rSS can show different clinical values in different laboratory inspections or tests combined with non-digital subtraction angiography (non-DSA) and can be employed for the assessment of the prognosis pertaining to non-coronary artery lesions. Notably, rSS has some limitations. With the deepening of clinical research and the enrichment of examination methods, more rSS-derived scores have been discovered and applied in clinical practice. This article summarizes and reviews the clinical application of rSS and its derived scores to optimally utilize them as a series of important tools (Fig. 1).

### 1.1 Residual SYNTAX Score (rSS)

The ideal goal of PCI is complete revascularization (CR) of all diseased segments. The significance of CR in predicting outcomes and incomplete revascularization (ICR) varies in different studies, possibly because of the absence of a widely acknowledged definition, variations in statistical and methodological approaches, and variations in study populations; as a result, different studies have conflicting results [4]. The anatomically accepted definition of ICR is the presence of at least one vessel larger than 2.0 mm in diameter with a minimum of at least one lesion in the coronoary artery after PCI exhibiting stenosis exceeding 50% (the standard for CABG treatment is 1.5 mm). There is a premise indicating that regardless of the location, complexity, or clinical background of the vascular lesion, ICR is defined as any lesion that has not been treated. Although CR can broadly improve myocardial ischemia and prevent unplanned revascularization, the overaggressive treatment of CR may lead to restenosis within the stent [5] and thrombosis of the stent [6], thus increasing the likelihood of complications during the perioperative period [7]. CR is frequently impractical to implement in individuals with multivessel CAD for various causes, including chronic total occlusion (CTO), severe calcification, significant impairment of the left ventricle’s function, or poor medical condition. PCI in individuals with lesions of greater complexity may lead to a rise in procedure-related adverse events. CR is not easily achieved in medical practice and may not improve the prognosis of patients; therefore, indiscriminately pursuing CR is needless. Reasonable revascularization is of great significance in guiding clinical decision-making.

**Fig. 2.**
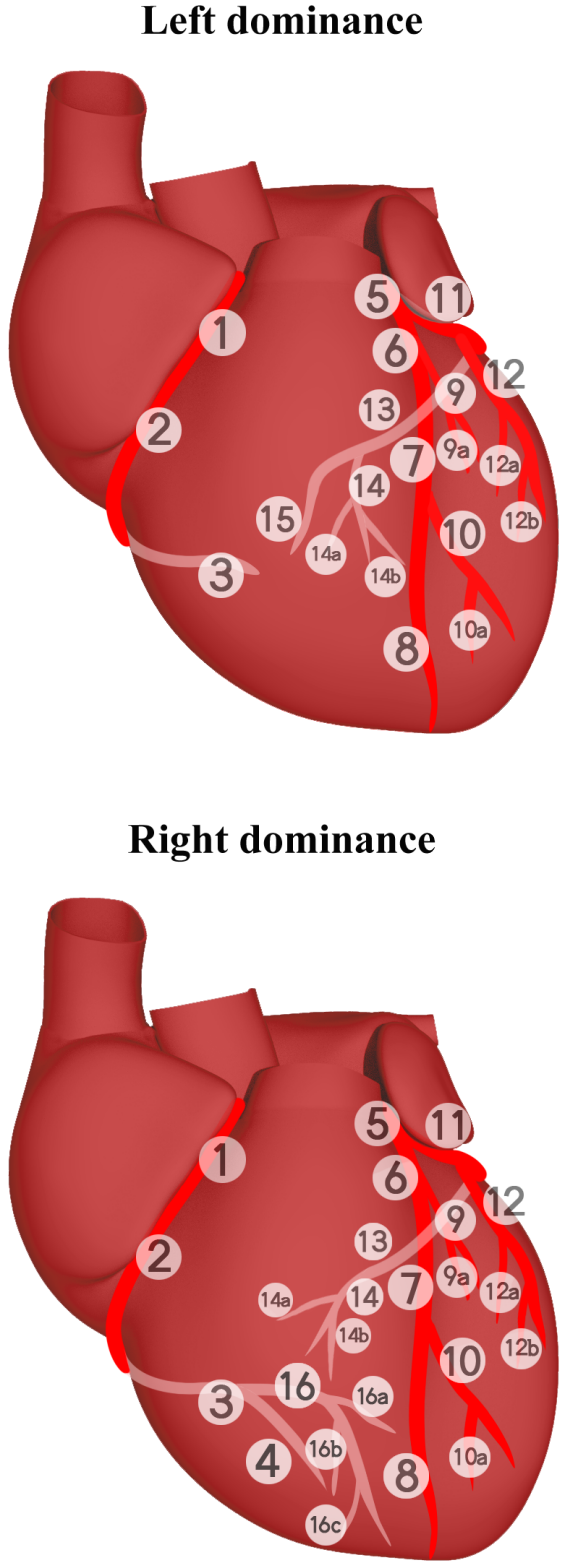
**Distribution of coronary arteries in the heart according to SYNTAX score (SS)**. The numbers in the figure represent the segments divided by SS and correspond to the following definition of segments: 1: RCA proximal; 2: RCA mid; 3: RCA distal; 4: Posterior descending artery; 5: Left main; 6: LAD proximal; 7: LAD mid; 8: LAD apical; 9: First diagonal; 9a: Additional first diagonal originating from segment 6 or 7, before segment 8; 10: Second diagonal; 10a: Additional second diagonal originating from segment 8; 11: Proximal circumflex artery; 12: Intermediate/ anterolateral artery; 12a: First side branch of circumflex running in general to the area of obtuse margin of the heart; 12b: Second additional branch of circumflex running in the same direction as 12; 13: Distal circumflex artery; 14: Left posterolateral; 14a: Distal from 14 and running in the same direction; 14b: Distal from 14 and 14 a and running in the same direction; 15: Posterior descending; 16: Posterolateral branch from RCA; 16a: First posterolateral branch from segment 16; 16b: Second posterolateral branch from segment 16; 16c: Third posterolateral branch from segment 16. The calculation of SS can be done at this latest website: https://syntaxscore.org/. SS, SYNTAX score; LAD, left anterior descending; RCA, right coronary artery.

SS is a commonly used tool for coronary artery assessment in clinical practice. SS can quantitatively evaluate the location, degree, and nature of coronary artery lesions according to their anatomical structure and can effectively and precisely assess their complexity and progression (Fig. 2) [3]. However, the application of SS cannot fully reflect the improvement of coronary artery lesions after PCI [8]. To solve this problem, rSS was developed after the Acute Catheterization and Urgent Intervention Triage Strategy (ACUITY) study [9]. rSS, defined as the residual SYNTAX score after PCI or CABG, is a score for lesions with >50% stenosis in vessels ≥1.5 mm in diameter. It can be used to quantitatively calculate the angiographic integrity and residual atherosclerotic burden of revascularization after PCI or CABG. Therefore, it can accurately and intuitively reflect the residual lesions and be supplemented to determine whether the rate of survival after PCI is better than that of CABG. Additionally, it can predict cardiovascular events after PCI [10, 11] and has significance for guiding the formulation or optimization of patient follow-up programs.

In a retrospective study on CTO and multivessel CAD in patients after PCI, Jang *et al*. [12] discovered that the cardiac mortality of patients with rSS ≤12 exhibited a notable decrease compared to the patient group with rSS >12, and the cardiac mortality was comparable to that of patients undergoing CABG. Witberg *et al*. [13] suggest that if the total rSS exceeds 8, then CABG should ideally be performed to optimize the clinical outcome. Their study indicated that in patients with left main CAD and three-vessel disease, quantitative estimates of the completeness of revascularization using rSS were better than qualitative estimates, such as the residual total occlusion or number of lesion vessels, in the risk stratification of long-term consequences with ICR. According to previous research [14], rSS >8 is linked with worse long-term consequences and every additional point in rSS corresponds to a 6% rise in the risk of death at 12 months. Patients with a higher rSS tend to be older and have a higher probability of hypertension, diabetes, and prior myocardial infarction (MI); furthermore, they have a higher probability of cardiac function Killip class III–IV performance requiring emergency CABG and intra-aortic balloon counterpulsation (IABP) [15]. Although previous studies have identified various target populations for clinical research and obtained numerous cut-off points for rSS, it should be noted that the rSS is merely an anatomical score and does not take into account the diverse demographic and clinical characteristics of each study population; similarly, the endpoint events do not remain consistent across different studies. Therefore, if these differences are disregarded in clinical applications, satisfactory results cannot always be guaranteed; in conclusion, the cut-off point of reasonable revascularization remains controversial.

### 1.2 Syntax Revascularization Index (SRI)

SRI, first proposed by Généreux *et al*. [16], was calculated as the ratio of the difference between the baseline SS (bSS) and rSS to the bSS (SRI = (bSS-rSS)/bSS × 100%) to evaluate the extent of revascularization in PCI. Previous studies showed SRI ≥70% was a reasonable target for revascularization in complex coronary artery lesions. A subsequent study [17] of 1851 patients with complex coronary revascularization reported the all-cause mortality rate of SRI ≥85% was similar to that of the CR group. However, the incidence of adverse outcomes, such as MACE, was significantly higher in the low-SRI group. Therefore, SRI ≥85% was a reasonable target for revascularization in this cohort. Additionally, Song *et al*. [18] found that SRI <70% and rSS ≥8 had a similar predictive ability for 2-year all-cause death, repeat revascularization, and MACE.

### 1.3 Clinical Residual Syntax Score (CRSS)

The limited inclusion of clinical parameters in the rSS poses a challenge to its effectiveness in accurately stratifying the risk of patients with complex CAD. rSS is a scoring system for angiography; CRSS is obtained by integrating clinical variables with rSS. CRSS is calculated by multiplying rSS by the modified age, creatinine, and ejection fraction score, i.e., the “modified age, creatinine, and ejection fraction (ACEF)” score [19]. Studies have shown that CRSS better predicts 1-year all-cause mortality and target lesion failure (TLF) rates in comparison to rSS alone, and patients with CRSS >12 have gradually increased adverse long-term outcomes. The ability of CRSS is similar to that of rSS to predict patient-oriented composite events (POCE). However, CRSS greatly enhances the ability to forecast secondary endpoints, such as death [20]. Yan *et al*. [21] extended the application of CRSS in patients with chronic kidney disease (CKD), and receiver operating characteristic (ROC) curve analysis suggested that CRSS showed higher predictive performance in all-cause and cardiac mortality and MACE. Notably, the predictive accuracy of rSS for unplanned revascularization (UR) is better than that of CRSS, which is similar to the study results of Song *et al*. [18]. Therefore, the progression of residual CAD is speculated to be the primary cause of UR. These findings are helpful for clinicians to evaluate the risks and long-term prognosis of patients with high CRSS and to develop diagnostic and treatment strategies.

### 1.4 Residual SS-II Score (rSS-II)

Focusing solely on anatomical factors overlooks individual differences caused by clinical factors, leading to false risk stratification. The SYNTAX II score (SS-II) is obtained by assessing clinical features which improve the ability to predict risk and combining them with SS to assess and compare long-term mortality between PCI and CABG strategies. Variables in the SS-II include peripheral arterial disease (PAD), chronic obstructive pulmonary disease (COPD), left main (LM) disease, left ventricular ejection fraction (LVEF), estimated glomerular filtration rate (eGFR) sex, and age [22]. A multi-ethnic minority cohort study has shown that additional modifications for other variables and comorbidities did not change the association’s magnitude in the SS-II score, suggesting that the comorbidities included in the scoring system are the most important [23]. Furthermore, when combined with clinical variables, rSS-II has a richer set of variables compared with CRSS, as it combines several clinical comorbidities closely related to prognosis, all of which are nearly irreplaceable. While certain factors in SS-II (age, sex, PAD, and COPD) remain the same after revascularization, other variables, such as CRCL, LVEF, anatomical SS, and unprotected LM stenosis, may improve or worsen after PCI. A prospective, multicenter cohort study showed that combining SS-II and rSS could help identify the increased risk of long-term clinical adverse events in patients with acute coronary syndrome (ACS) and multivessel disease (MVD). In Cox regression analysis, rSS-II exhibited a correlation with mortality from all causes during a 5-year follow-up period and better stratified the risk of all-cause mortality and MACE than rSS [24]. In a study [23] of patients with three-vessel or LM disease who underwent initial PCI for ST-segment elevation MI (STEMI) during long-term follow-up (mean: 4.9 years), higher rSS-II scores were linked to a higher likelihood of mortality and readmission. This study supports the utility of rSS-II to guide the risk stratification and revascularization strategy selection in STEMI patients with LM disease or MVD.

### 1.5 Modified Residual Syntax Score (mrSS)

Previous studies have shown that the revascularization of diseased segments only with a diameter ≥2.5 mm is similar to achieving revascularization in the context of the long-term prognosis [25]. PCI in small vessel disease which may cause stent thrombosis and bleeding resulting from the prolonged use of dual antiplatelet therapy (DAPT), and only one-third of small vessel lesions are functionally significant; the remaining vessels may be sufficient to ensure myocardial perfusion [26]. The findings that 35% of lesions with 50–70% stenosis are functionally significant [27] and that staged PCI of these lesions has good long-term outcomes [28] suggest that PCI for small or moderate lesions is not a priority. Park *et al*. [29] developed a modified rSS (mrSS), calculated by adding the scores for lesions with stenosis of at least 70% in each vessel with a diameter of 2.5 mm or more after PCI. It is easier to calculate the mrSS than the rSS after ignoring lesions with <70% stenosis or vessel diameters <2.5 mm. They found that patients who underwent reasonable ICR (R-ICR; rSS >0, but mrSS = 0) had similar clinical outcomes as those who underwent CR (rSS = 0) and that the results were superior to those of ICR (mrSS >0). These findings indicate that PCI for small vessel disease or intermediate disease can be safely delayed and that revascularization only for lesions with ≥70% stenosis in vessels ≥2.5 mm in diameter is reasonable in patients with MVD. R-ICR strategies based on mrSS scores reduce the residual ischemic burden, prevent surgery-related complications compared to CR strategies, and are more convenient to use.

### 1.6 Fractional Flow Reserve (FFR) and Residual Functional Syntax Score (rFSS)

**Fig. 3.**
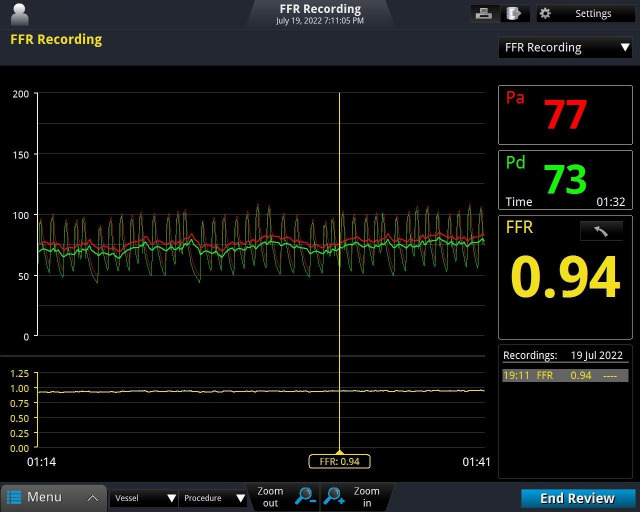
**Real-time recording of fractional flow reserve (FFR)**. In the selected segment of the occluded vessel, Pd and Pa are the distal and proximal blood pressure values of the vesse, and FFR is equal to PD divided by PA. Pd is the aortic pressure measured by the guide catheter and Pa is the distal pressure measured by the pressure guide wire. FFR, fractional flow reserve.

The anatomic lesion severity was inconsistent with the functional significance based on FFR [27, 30, 31]. FFR is considered the “gold standard” for functionally assessing the severity of ischemia caused by coronary artery stenosis and guiding revascularization procedures (Fig. 3). An FFR-guided revascularization strategy is more rational than angiography-guided revascularization or medical therapy [32, 33]; once functional CR (FCR) is achieved, the results are likely to be similar, regardless of the anatomy of the residual disease [34]. Previously, Nam *et al*. [35] developed the functional SYNTAX score (FSS) concept by integrating SS and FFR; they calculated SS only in vessels with low FFR (FFR <0.8) and showed that FSS predicted clinical outcomes better than SS. A previous study [36] indicated that despite the achievement of successful PCI through angiography, approximately 20% of patients had an FFR of 0.80 or less. In a study of 1910 patients with FFR >0.80 after PCI, Lee *et al*. [37] showed that FFR had a strong correlation with the incidence of target vessel failure (TVF) during the 2 years of study. However, there were no notable disparities observed in TVF at 2 years among the rSS categories. This study suggests that FFR after PCI is a more effective method for evaluating the remaining atherosclerotic burden compared to angiographic evaluation.

rFSS is the cumulative sum of residual scores for vessels exhibiting low FFR (FFR <0.80); it can provide comprehensive anatomical and functional information regarding the remaining disease burden following PCI. The 3V-FFR-FRIENDS [38] (i.e., three-vessel fractional flow reserve for the assessment of total stenosis burden and its clinical impact in patients with coronary artery disease) study was a prospective, multicenter, observational study. Its substudy [39] involved 385 patients undergoing PCI for poor vascular function. The study compared 3-vessel FFR, rSS and rFSS. Although all three scoring systems showed notable connections with the risk of MACE at 2 years, when the three scoring systems were separately combined with clinical risk factors, the model with rFSS showed the highest discriminatory function (C index) for MACE at 2 years, and only rFSS improved the discriminatory function for MACE. In addition, this study showed that patients with functional IR (FIR; rFSS ≥1) defined by FFR based on rFSS had significantly higher 2-year MACE rates than those with FCR (rFSS = 0). These findings imply that the integration of anatomical and functional data yields a more accurate evaluation of patient risk following PCI compared to relying solely on either anatomical or functional evaluations.

### 1.7 Quantitative Flow Ratio (QFR) and rFSS

**Fig. 4.**
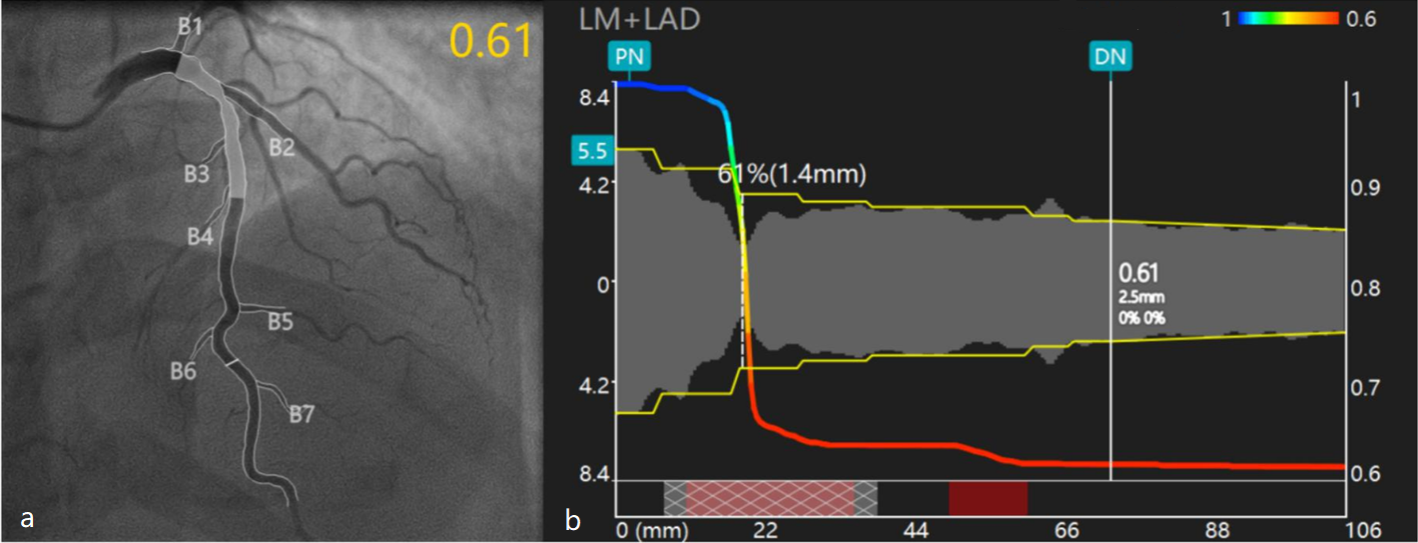
**Quantitative flow ratio (QFR) analysis based on coronary angiography**. (a) After QFR analysis, the gray part is the recommended placement of the virtual stent. (b) The QFR at the location of the vessel intercepted by the white vertical line is 0.61. PN and DN are the two normal points of the selected blood vessels as reference points. When the white tangential line of the blood vessel selected in (a) continues to move to the distal end of the blood vessel, the white vertical line in (b) continues to move to the right and the QFR of the selected blood vessel will be analyzed in real time. B1–B7 are small branches from LM to LAD. QFR, quantitative flow ratio; DN, distal normal; LM, left main; LAD, left anterior descending; PN, proximal normal.

Although FFR is the gold standard for coronary physiological assessments, its utilization is significantly less extensive than expected, possibly due to the cost of pressure guidewires, additional procedures, prolonged procedural times, and side effects caused by adenosine. To overcome these real-world clinical limitations and further expand the practical scope of physiological lesion assessments, wire-free QFR based on coronary angiography was developed as a new tool for coronary physiological assessment. QFR is a reliable and rapid method to calculate functional parameters based on three-dimensional quantitative coronary angiography to detect hemodynamically significant lesions. The pressure curve is simulated from the angiography images by computer software, and the value of QFR is calculated according to the pressure difference between the two ends of the selected lumen (Fig. 4). Compared with FFR, QFR calculated only based on imaging has the advantages of not using hyperemia-inducing drugs and pressure guidewires, short operation times, and specific clinical diagnostic accuracy. The FAVOR pilot [40] study showed that QFR and FFR measurement results are consistent; the FAVOR II China [41] study was the first to reveal the diagnostic accuracy of real-time online QFR analysis in the catheterization laboratory. With FFR as the reference standard, the final QFR diagnostic accuracy was 92.7%, consistent with the FAVOR II Europe-Japan study [42]. Furthermore, the latter suggested that QFR could reduce the procedure time by 28% compared with FFR. These studies show that QFR technology is practical and reliable in clinical practice. Tang *et al*. [43] combined QFR with rSS and discarded smaller vessels (<1.5 mm) that were not functionally important and had uncertain revascularization benefits in traditional SYNTAX; rSS was measured in coronary arteries with QFR ≤0.80 in vessels with diameters >2 mm. The results of the QFR-guided residual functional SYNTAX score (Q-rFSS) showed that the risk of MACE in patients with FIR (Q-rFSS ≥1) was significantly higher in patients with FCR (Q-rFSS = 0). Moreover, Q-rFSS has improved discriminatory power for clinical outcomes compared with anatomical rSS.

In the study by Lee *et al*. [44], patients who attained FCR as determined by rFSS experienced a notable improvement in exercise duration following PCI, in contrast to patients with incomplete or partial FIR, similar to the results obtained by Xue *et al*. [45] using rSS; this study compared patients’ exercise time, while Xue *et al*. [45] compared cardiopulmonary exercise testing (CPET) variables. However, the study by Lee *et al*. [44] showed that an increased exercise time correlated more strongly with rFSS than with rSS, a drop in SS, or an increase in three-vessel QFR. Among the parameters of post-PCI anatomical or functional outcomes, rFSS is superior in predicting the post-PCI exercise capacity or clinical outcome.

The FAVOR III China Trial [46] was a prospective, multicenter, blinded, randomized clinical trial of 3830 patients divided into QFR- and coronary angiography-guided groups on a 1:1 basis. PCI was performed for 50–90% stenosis of arterial diameters ≥2.5 mm; the primary endpoint was MACE at 1 year after PCI. An analysis of that study [47] suggested that approximately two-thirds of patients diagnosed with angiographic IR were reclassified as having FCR (rFSS = 0) following the utilization of QFR. Additionally, there was no notable disparity in the prognosis between these patients and other FCR patients, which aligned with the findings of a prior study [34]. QFR guidance can prevent unnecessary stent implantation in lesions with a good prognosis and screen out nonobstructive lesions with a poor prognosis, which ultimately affects the revascularization plan of about a quarter of patients and improves the clinical prognosis of these beneficiaries at 1 year; this suggests that using rFSS provides a better risk stratification ability and clinical prognosis than dissecting rSS and clinical variables alone.

## 2. rSS and Examination Methods

### 2.1 Coronary Computed Tomography Angiography (CCTA) and Exercise Electrocardiogram (ECG)

**Fig. 5.**
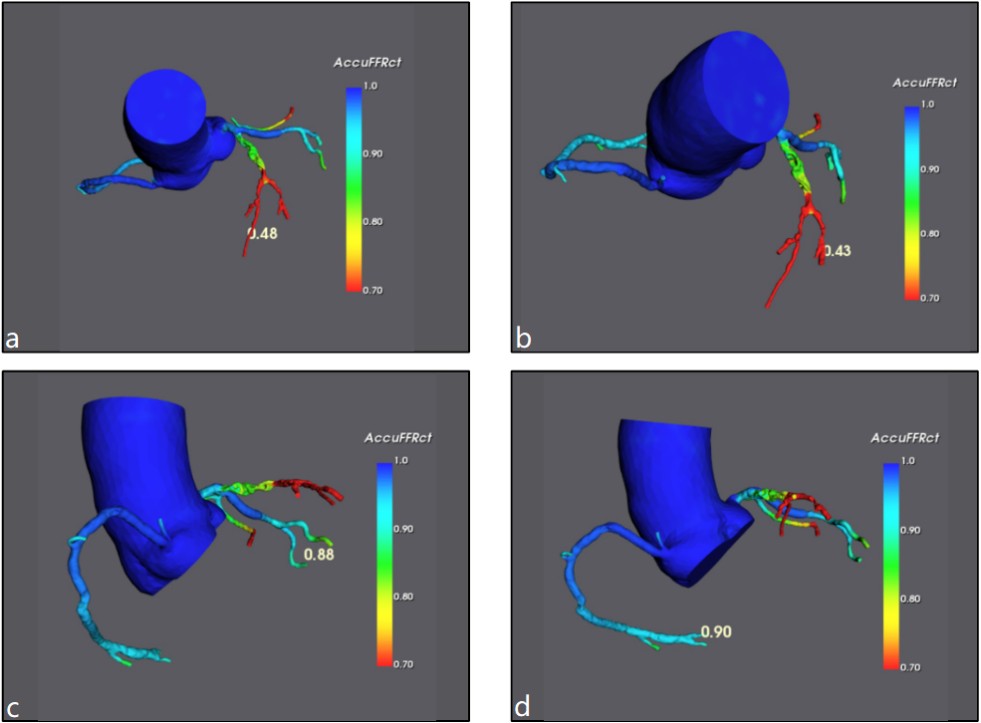
**Functional analysis of coronary computed tomography angiography (CCTA)**. (a–d) The four images represent the functional analysis of different parts of the coronary artery. (a) and (b) analyze the different branches of the left anterior descending. The FFR obtained by the three vessels is as follows: LAD: 0.48 (a) and 0.43 (b) ; LCX: 0.88 (c); RCA: 0.90 (d) . LAD, left anterior descendin; LCX, left circumflex (branch); RCA, right coronary artery; CCTA, coronary computed tomography angiography; FFR, fractional flow reserve.

Zhang *et al*. [48] conducted noninvasive examinations, including exercise ECG and CCTA, combined with rSS separately for a retrospective study. As the limitations of FFR have been described previously, the computational pressure-flow dynamics-derived FFR (caFFR) was used as the reference standard instead of invasive FFR (Fig. 5). Patients older than 60 years of age were enrolled and divided into caFFR-positive (≤0.80) and caFFR-negative (>0.80) groups. The findings indicated that there was no notable disparity observed in ECGs between the two cohorts, but the caFFR-positive group exhibited a significantly elevated rSS. Combined with typical symptoms, the utilization of rSS in conjunction with CCTA has the potential to enhance the detection sensitivity and precision of diagnosing myocardial ischemia, and is feasible and safe. Although the diagnostic performance of nuclear myocardial perfusion imaging and adenosine-stress myocardial perfusion assessed through CT is significantly better than that of exercise ECG and CCTA, applying these methods has several limitations [49, 50, 51]. Therefore, integrating rSS with symptoms and CCTA could be beneficial in clinical practice. However, the cost of the disposable sensor used with caFFR in the Zhang *et al*. [48] study should be considered. Notably, the accuracy of CCTA is affected by the image quality and stents, which often affect the clinician’s judgment due to artifacts and image defects [52]; therefore, improving the resolution can enhance diagnostic accuracy.

### 2.2 Optical Coherence Tomography (OCT) and Intravascular Ultrasound (IVUS)

Wang *et al*. [53] studied the prognostic impact of rSS and the culprit plaque morphology on MACE in 274 patients with STEMI. The study was divided into two aspects, and patients were divided into four groups based on rSS and plaque morphology, including plaque rupture (PR)/high rSS, PR/low rSS, plaque erosion (PE)/high rSS, and PE/low rSS. Patients’ plaques were analyzed by OCT, and patients were divided into four groups (according to high-risk plaques (HRP), defined by OCT [54] combined with rSS), including HRP/high rSS, HRP/high rSS, non-HRP/high rSS, and non-HRP/low rSS. The study showed that patients with PR/high rSS had a higher risk of plaques and a 4.80-fold higher risk of cardiovascular events than those with PE/low rSS; however, patients with HRP/high rSS had a higher risk of MACE. Furthermore, the author stated in another article [55] that patients with higher rSS (rSS >8) had a higher incidence of PR and HRP. rSS assesses the vascular anatomy rather than plaque characteristics; therefore, including the culprit plaque morphology and OCT evaluation in the angiographic evaluation of rSS may provide a better risk prediction ability.

In addition, Fujino *et al*. [56], in the Providing Regional Observations to Study Predictors of Events in the Coronary Tree (PROSPECT) study [57], assessed the plaque morphology by greyscale IVUS and virtual histology IVUS (VH-IVUS) and discovered an association with rSS. The results suggest that the presence of ≥1 lesions with a plaque burden ≥70% or ≥1 VH thin-cap fibroatheroma (VH-TCFA) improves the predictive power of non-culprit-related MACE in addition to that of rSS and clinical factors. Compared with angiographic evaluations, a morphological evaluation by IVUS may help identify high-risk populations.

### 2.3 Myocardial Perfusion Single-Photon-Emission Computed Tomography (SPECT)

The application of SPECT enables the prediction of adverse cardiac events in patients with CAD [58]. The difference in the percentage of the total myocardium Safety Data Sheet (SDS%) between the first and second SPECT—ΔSDS% was used to assess the ischemic improvement. In a study by Hayase *et al*. [59], the risk of MACE was stratified by a combination of ΔSDS% and rSS. rSS does not solely reflect the quantitative ischemic reduction, but its combination with ΔSDS% can accurately predict MACE after revascularization; the risk of MACE was stratified according to ΔSDS% and rSS. Using ROC analysis, the best cutoff value of rSS was estimated to be 12. Patients with low rSS (<12) and a 5% reduction in ischemia after revascularization had the best prognosis, while patients with high rSS (≥12) and no significant improvement in ischemia (ΔSDS% <5%) had the worst prognosis. Compared with rSS alone, combining ΔSDS% and rSS significantly improved the accuracy of predicting MACE.

## 3. rSS and Noncoronary Lesion Prognosis

### 3.1 CKD and Contrast-Induced Nephropathy (CIN)

Patients with CAD have a higher likelihood of experiencing complications with CKD, and patients with CKD face a significant risk factor of cardiovascular disease [60]. Once patients with cardiovascular disease are complicated with CKD, they may have more complex anatomical problems such as calcification, bifurcations, long lesions, and multi-vascular diseases, which will increase the related complications of surgery, reduce the success rate of surgery, and lead to higher mortality [61]. In addition, patients with CKD are more likely to develop CIN after PCI [62]; therefore, ICR treatment may be more appropriate for patients with complex CKD undergoing PCI to reduce the potential surgical risk.

Yan *et al*. [63] used rSS as a quantitative tool to evaluate the degree of ICR in patients with CKD. In this study, subjects were divided into the CR group (rSS = 0), R-ICR group (0 < rSS ≤ 8), and ICR group (rSS >8) according to rSS values. The R-ICR and CR groups had comparable hazards of all-cause death, cardiac death, MI, and stroke, and rSS was more accurate than basic SS in predicting the risk of unplanned revascularization, stroke, and MACE according to the ROC curve analysis, which may provide some guidance for interventional therapy.

Cardi *et al*. [64] have shown that patients with CKD have higher rSS values; studies have found that patients with rSS >8 have a higher incidence of acute renal failure [15]. To avoid the effect of an LVEF reduction on the incidence of CIN, Kucukosmanoglu *et al*. [65] selected study subjects from non-STEMI (NSTEMI) patients with normal or nearly normal LVEF and discovered that patients with high rSS values had a higher incidence of CIN. Notably, the cutoff value of rSS was 3.5, with a sensitivity of 79% and a specificity of 65% for CIN. However, in STEMI patients, rSS has been proven to be an independent predictor of CIN development [66].

### 3.2 Exercise Tolerance

Xue *et al*. [45] conducted a retrospective study to quantify ICR indexes by rSS and evaluated the impact of ICR on exercise tolerance. A total of 87 patients underwent CPET within a year following PCI; CPET variables were collected and compared. According to the rSS, the patients were divided into the CR (rSS = 0), R-ICR (0 < rSS < 8), and severe ICR (sICR; severe residual lesion of the coronary artery, rSS >8) groups. The study showed no significant difference in CPET variables between the CR and R-ICR groups. However, in the sICR group, the CPET variables gradually decreased with an increase in rSS values, indicating a decrease in exercise tolerance. Studies have shown that R-ICR is a reasonable and safe degree of revascularization in patients with CAD. But when residual CAD burden exceeds this threshold and loses compensation, the long-term adverse clinical outcomes, including adverse exercise tolerance, will significantly increase.

### 3.3 Influence on the Prognosis of Transcatheter Aortic Valve Replacement (TAVR)

CAD exists in more than half of the population with TAVR [67, 68]. Early trials reported no association between CAD and increased mortality after TAVR [69, 70]; however, later trials used SS to stratify the CAD severity, resulting in conflicting conclusions [71]. Witberg *et al*. [72] reviewed six studies using rSS to define R-ICR and ICR thresholds in 3107 patients and conducted a meta-analysis of the prognosis of TAVR with a follow-up period of 0.7–3 years. The results showed that patients with CAD who had R-ICR (0 < rSS < 8) before TAVR had a mortality risk equivalent to that of patients without CAD; however, those who had an unreasonable ICR (rSS >8) had a mortality risk that was approximately tripled, suggesting that appropriate pre-TAVR revascularization may improve outcomes in patients undergoing TAVR, and adequate revascularization compared with treating other adverse conditions, such as atrial fibrillation, obstructive lung disease, renal function, and frailty, is an easier intervention before TAVR [73, 74]. This study suggests that as previously measured by SS, the extent of CAD is not directly related to mortality; conversely, appropriate and timely revascularization, as assessed by rSS, is most strongly associated with mortality risk, which may be another advantage of rSS over SS.

Notably, a subsequent study [75] did not show an association between CAD and the degree of revascularization after TAVR, during long-term follow-up. Therefore, Scarsini *et al*. [76] considered the anatomy alone and in combination with the function and proved that incomplete functional revascularization was associated with poor clinical outcomes after TAVR through rFSS, a derivative of rSS. However, the sample size in the study was limited, and further studies are needed for confirmation.

### 3.4 Guiding DAPT

DAPT with a P2Y12-receptor inhibitor plus aspirin is essential for preventing coronary stent thrombosis. The prospective, multicenter ILOVE-IT2 trial [77], in which all patients continued to take a minimum of 75 mg of clopidogrel and 100 mg of aspirin for 6 or 12 months after stent implantation, was investigated for composite clinical endpoints. A secondary analysis of this trial showed that in the low rSS group (rSS = 0), patients who used DAPT for 6 months after PCI were not inferior to the subgroup that continued DAPT for 12 months. In contrast, patients at higher risk after PCI (rSS >0) were not inferior to the subgroup that continued DAPT for 12 months. Only 6 months of DAPT may have contributed to the net adverse clinical events (NACE) and patient-oriented composite endpoint (PoCE) at 6–12 months; the long-term benefit of DAPT with a high rSS may be greater. rSS did not significantly differ in bleeding events at 12 months among patients who discontinued DAPT at 6 months. For clinicians, bleeding events due to the use of antiplatelet drugs after PCI and ischemic events due to insufficient antiplatelet drugs are vexing contradictions and are not uncommon in clinical practice. Although guidelines [78, 79] recommend that the standard duration of DAPT after PCI be at least 6–12 months, there is great individual variation in patients, especially in high-risk groups, and the optimal timing of antiplatelet therapy should be determined after a full evaluation of hemorrhage and ischemic risks. Additionally, the guidelines recommend using bleeding and ischemia scores to assist in deciding on antiplatelet therapy. However, anatomical methods still exist to quantify the risk of ischemia. For patients with high bleeding and low ischemic risks, a shorter DAPT duration can be considered, and for patients with low bleeding and high ischemic risks, a longer DAPT duration may be considered. rSS can potentially be an essential tool for assessing the ischemic risk and may play an important role in guiding the use of DAPT.

## 4. Predictors of rSS

### 4.1 CHA2DS2-VASC Score

The CHA2DS2-VASc score is used to evaluate patients with cardiovascular disease [80] and atrial fibrillation to assess the risk of thromboembolism [81] and is increasingly widely used in various scenarios. The coronary thrombotic state is related to the extent of stenosis in coronary arteries, and research has indicated that this score can predict adverse events after ACS [82]. In a novel study [83] involving 688 STEMI patients after PCI, a positive correlation between the rSS and CHA2DS2-VASc score was confirmed for the first time, and the CHA2DS2-VASc score exhibited a strong ability to forecast elevated rSS (rSS >8).

### 4.2 Triglyceride-Glucose (TYG) Index

Type 2 diabetes mellitus (T2DM) is an important contributing factor to the development of CAD. The TyG index is derived from the product of fasting blood glucose and triglyceride levels and can be employed for evaluating the risk of cardiovascular disease [84]. In previous studies [85, 86, 87], the TyG index has shown a strong predictive power for the post-PCI risk in different cohorts; however, none combined the TyG index with rSS. Xiong *et al*. [88] investigated the prognostic value of adverse cardiac consequences in T2DM patients after PCI and the possible added predictive significance of combining rSS with the TyG index. The results showed poor outcomes after PCI were more prevalent in patients with T2DM with rSS >7.5 than in those with rSS ≤7.5. Additionally, the results demonstrated, for the first time, that combining rSS and the TyG index could improve the predictive ability for patients with DM for detecting patients at an elevated risk level after PCI and creating personalized care strategies to improve clinical outcomes.

### 4.3 Neutrophil to Lymphocyte Ratio (NLR)

Inflammation has a crucial function in the pathophysiology of CAD. Factors such as the vascular damage caused by increased neutrophil activity, activation of coagulation pathways, microvascular obstruction (caused by platelet aggregation), and myocardial cell necrosis (caused by proinflammatory cytokine secretion), lead to an increased risk of thrombosis and plaque rupture [89]. In addition, neutrophils have connections to escalate blood viscosity, hypercoagulability, and the induction of microvascular and reperfusion injury [90]. Furthermore, the cortisol secretion induced by the stress associated with ACS leads to increased lymphocyte apoptosis, and the inflammatory response induces lymphopenia. NLR serves as a predictive factor linked to the inflammatory condition of CAD. It has demonstrated superior predictive capability compared to neutrophil or lymphocyte counts and exhibited independent prognostic value for high rSS in 613 patients with STEMI. It has shown to be a better prognostic factor than the neutrophil or lymphocyte count and was an independent predictor of high rSS in 613 patients with STEMI [91]. Notably, this study suggests that diabetes is closely related to increased inflammation, and diabetic patients have higher NLR, which is a potential risk of high rSS. In addition, NLR is positively associated with age, which can lead to an increased coronary burden. LVEF-related coronary atherosclerosis may lead to systemic inflammation, resulting in a higher NLR and adversely affecting the blood vessels; LVEF is negatively correlated with NLR. These factors can affect residual CAD and increase the risk of ischemic occurrences.

### 4.4 White Blood Cell Count to Mean Platelet Volume Ratio (WMR)

White blood cells play a crucial role in the progression of atherosclerosis and instability, and have the potential to result in thrombotic occurrences. An increased white blood cell count (WBC) has been linked to higher mortality rates in patients with STEMI. The mean platelet volume (MPV) serves as an extremely responsive indicator for platelet activity, and larger platelets have higher thrombotic potential. The changes in MPV and WBC levels caused by increased inflammation and thrombosis could elucidate the elevated WMR levels in patients with ACS. The advantage of WMR is that it is performed at no additional cost in high-risk clinical situations such as STEMI. However, its ability to accurately assess risk and its potential for enhancing clinical risk categorization and treatment strategizing makes it an excellent tool. WMR is more efficient than other whole blood cell indices in predicting long-term MACE in patients with NSTEMI. Patients with high WMR (≥1286) have also demonstrated higher rSS and increased MACE, which may be related to the interaction between inflammation and residual CAD [92].

## 5. Conclusions

After over 10 years of development, rSS is increasingly used in clinical practice. It is suitable for evaluating complex vascular diseases and can be combined with various clinical tools to predict the prognosis of patients and guide treatment. However, when performing rSS-related calculations, especially the calculation of the corresponding weights of each blood vessel of the patient, the help of calculation tools or websites is required or the weight table is consulted for manual calculation, unless that person is an expert in this field. In addition, some hospitals in the real world, especially primary hospitals, may focus mainly on solving the difficulties encountered on the operating table and the immediate effect of the operation, rather than paying more attention to how to improve the long-term prognosis, so the popularity of this score is limited. One solution is that the rSS-related calculation can be directly implanted into the angiography imaging device through the designed script, and the artificial intelligence can automatically and objectively analyze each blood vessel diameter and blood vessel condition and calculate the corresponding score immediately, or even can be directly implanted into the instrument to calculate QFR, and directly obtain the rSS-related score with or without function.

Compared with rSS, the derived scores have advantages suitable for various clinical settings. Compared with the standard anatomical assessment of coronary angiography, QFR technology has a superior specificity and sensitivity and is more efficient and safer than traditional FFR. In addition, it has clinical benefits after achieving FCR. Due to these advantages, QFR may become an essential tool for interventional therapy; however, the available evidence for the use of QFR in evidence-based medicine is still relatively limited. With the advancement of various clinical studies, the role of QFR may become increasingly important, and QFR-based rFSS has broad application prospects.

However, there are still some limitations with Q-rFSS. First, although the scoring system includes a weighting factor for each lesion to distinguish the anatomical importance differences of each lesion, previous studies have suggested that atherosclerosis progresses faster in the proximal left anterior descending (pLAD) than in other segments [93], and pLAD coronary artery lesions possess greater predictive significance [94]. Futhermore, a study [95] has shown that rSS combined with residual pLAD outperforms rSS alone in predictive performance; therefore, a higher score weight may be required for pLAD coronary artery stenosis. Second, Q-rFSS only includes anatomical and functional evaluations and does not include clinical factors. Whether adding clinical factors, such as rSS-II, can improve the discriminative power of clinical results remains unclear. In addition, relevant studies have not confirmed whether including certain laboratory tests or auxiliary scores (as mentioned above) related to Q-rFSS can increase the predictive accuracy. Therefore, rSS and its derived scores can be developed and improved to be extensively used in various clinical scenarios.

## Abbreviations

3V-FFR-FRIENDS, three-vessel fractional flow reserve for the assessment of total stenosis burden and its clinical impact in patients with coronary artery disease; ACEF, age, creatinine, and ejection fraction; ACS, acute coronary syndrome; ACUITY, Acute Catheterization and Urgent Intervention Triage Strategy; bSS, baseline SYNTAX score; CABG, coronary artery bypass graft; CAD, coronary artery disease; caFFR, computational pressure-flow dynamics-derived fractional flow reserve; CCTA, coronary computed tomography angiography; CIN, contrast-induced nephropathy; CKD, chronic kidney disease; COPD, chronic obstructive pulmonary disease; CPET, cardiopulmonary exercise testing; CR, complete revascularization; CRCL, creatinine clearance; CRSS, clinical residual SYNTAX score; CTO, chronic total occlusion; DAPT, dual antiplatelet therapy; DSA, digital subtraction angiography; ECG, electrocardiogram; eGFR, estimated glomerular filtration rate; FCR, functional complete revascularization; FIR, functional IR; FFR, fractional flow reserve; FSS, functional SYNTAX score; HRP, high-risk plaques; ICR, incomplete revascularization; IVUS, intravascular ultrasound; LM, left main; LVEF, left ventricular ejection fraction; MACE, major adverse cardiovascular event; MI, myocardial infarction; MPV, mean platelet volume; mrSS, modified residual SYNTAX score; MVD, multivessel disease; NACE, net adverse clinical events; NLR, neutrophil to lymphocyte ratio; NSTEMI, non-ST-segment elevation myocardial infarction; OCT, optical coherence tomography; PAD, peripheral artery disease; PCI, percutaneous coronary intervention; PE, plaque erosion; pLAD, proximal left anterior descending; PoCE, patient-oriented composite endpoint; POCE, patient-oriented composite events; PR, plaque rupture; PROPSPECT, Providing Regional Observations to Study Predictors of Events in the Coronary Tree; QFR, quantitative flow ratio; Q-rFSS, QFR-guided residual functional SYNTAX score; rFSS, residual functional SYNTAX score; R-ICR, reasonable incomplete revascularization; ROC, receiver operating characteristic; rSS, residual SYNTAX score; rSS-II, residual SYNTAX score-II; SDS%, percentage of the total myocardium Safety Data Sheet; SICR, severe incomplete revascularization; SPECT, single-photon-emission computed tomography; SRI, SYNTAX revascularization index; SS, SYNTAX score; SS-II, SYNTAX score-II; STEMI, ST-segment elevation myocardial infarction; T2DM, type 2 diabetes mellitus; TAVR, transcatheter aortic valve replacement; TLF, target lesion failure; TVF, target vessel failure; TyG, triglyceride-glucose; UR, unplanned revascularization; VH-IVUS, virtual histology intravascular ultrasound; VH-TCFA, virtual histology thin-cap fibroatheroma; WBC, white blood cell count; WMR, white blood cell count to mean platelet volume ratio.
